# Emerging Innovations in Preoperative Planning and Motion Analysis in Orthopedic Surgery

**DOI:** 10.3390/diagnostics14131321

**Published:** 2024-06-21

**Authors:** Julien Berhouet, Ramy Samargandi

**Affiliations:** 1 Service de Chirurgie Orthopédique et Traumatologique, Centre Hospitalier Régional Universitaire (CHRU) de Tours, 1C Avenue de la République, 37170 Chambray-les-Tours, France; 2 Equipe Reconnaissance de Forme et Analyse de l’Image, Laboratoire d’Informatique Fondamentale et Appliquée de Tours EA6300, Ecole d’Ingénieurs Polytechnique Universitaire de Tours, Université de Tours, 64 Avenue Portalis, 37200 Tours, France; 3 Department of Orthopedic Surgery, Faculty of Medicine, University of Jeddah, Jeddah 23218, Saudi Arabia

**Keywords:** preoperative planning, accuracy, motion analysis, prediction, artificial intelligence, 3D level of evidence: V, expert opinion

## Abstract

In recent years, preoperative planning has undergone significant advancements, with a dual focus: improving the accuracy of implant placement and enhancing the prediction of functional outcomes. These breakthroughs have been made possible through the development of advanced processing methods for 3D preoperative images. These methods not only offer novel visualization techniques but can also be seamlessly integrated into computer-aided design models. Additionally, the refinement of motion capture systems has played a pivotal role in this progress. These “markerless” systems are more straightforward to implement and facilitate easier data analysis. Simultaneously, the emergence of machine learning algorithms, utilizing artificial intelligence, has enabled the amalgamation of anatomical and functional data, leading to highly personalized preoperative plans for patients. The shift in preoperative planning from 2D towards 3D, from static to dynamic, is closely linked to technological advances, which will be described in this instructional review. Finally, the concept of 4D planning, encompassing periarticular soft tissues, will be introduced as a forward-looking development in the field of orthopedic surgery.

## 1. Introduction

Preoperative planning (PP) is an essential step before any surgical procedure, providing a structured approach to optimize patient outcomes. PP has undergone significant advancements, with a dual focus: improving the accuracy of implant placement and enhancing the prediction of functional outcomes. With advancements in technology, the potential benefits of improved PP have become even more pronounced. Enhanced PP techniques, such as 3D modeling and templating, offer high precision in predicting implant sizes and positions, leading to a better alignment, fit, and function of surgical implants. These improvements not only ease the surgical process but also contribute to better postoperative recovery and long-term results.

Incorporating a robust PP process, especially with the integration of advanced imaging and planning tools, enables surgeons to anticipate and mitigate potential challenges before entering the operating room. This proactive approach is crucial for complex surgeries, where meticulous planning can mean the difference between success and complications.

Technological advances, such as computer-assisted orthopedic surgery (CAOS) [1,2,3,4] (i.e., navigation, robotics) and, more recently, mixed reality [5,6], mean that PP has become even more important. In practice, these technologies support the transfer of information generated during PP, and its subsequent execution, with the aim of being as accurate and reproducible as possible.

Thus, both the content and form of PP have evolved. Initially performed in a two-dimensional (2D) coordinate system, it is now based on three-dimensional (3D) reconstruction of the patient’s anatomy and the orthopedic implants [7,8]. While it was initially limited to static information about the implant’s position, PP now incorporates dynamic, functional, and increasingly personalized objectives, in part due to advances in the various computerized tools available (image processing, computer-aided design [CAD], artificial intelligence [AI], etc.). But, PP will only continue to evolve if the patients’ functional outcomes are rigorously evaluated in clinical practice.

This instructional review aims to summarize the current methods and complexities of PP and motion analysis as a means to introduce the concept of “predictive functional 4D planning”. I intend to answer four questions:

What does modern preoperative planning consist of?

What does modern motion analysis consist of?

How can preoperative planning and functional movement analysis be connected?

What is still missing to achieve predictive functional 4D planning?

## 2. What Does Modern Preoperative Planning Consist of?

### 2.1. Principles and Objectives of Preoperative Planning

According to the Cambridge Dictionary, the verb plan means “to make careful and detailed arrangements for the different stages of a process or event”. By extension, to plan also means to foresee or anticipate, i.e., “to think about and decide on a method for doing or achieving something”.

Thus, for a surgeon, PP consists of establishing an appropriate strategy for the chosen surgical intervention for a given patient. PP includes not only the surgical procedure but also encompasses several critical components. These components range from necessary calculations, such as determining anatomical measurements and selecting the size and positioning of implants, to designing surgical templates. The role of PP has been significantly enhanced using advanced imaging processing, image reconstruction, and 3D rendering software packages, which assist in creating detailed visualizations and simulations of the planned procedure. However, these technological advances do not replace the intellectual process that one goes through when preparing for surgery. Clinical reasoning is the basis for the diagnosis and, subsequently, for choosing the appropriate surgical indication. PP must take into consideration the patient’s symptom history, the physical examination and the imaging findings. It must identify the functional requirements that led to the consultation. PP also includes a thorough assessment of the technical means and materials needed for the surgery, considering their availability and the potential surgical challenges that may arise. At the end of this intellectual process, PP must lead to validation of the initial surgical indication, or to it being revised if the planned surgical procedure cannot fully meet the objectives that were fixed.

The term “planning accuracy” in the context of PP is multifaceted. It reflects the thoroughness and correctness of the surgeon’s analysis of the clinical situation and the formulation of the surgical strategy. However, PP does not guarantee that the surgical procedure will be carried out precisely from a technical standpoint, nor that the planned and desired outcomes will be achieved fully [9,10,11]. This surgical precision stems from the intraoperative CAOS tools that will be used (navigation, patient-specific guides, augmented reality [AR]) once the planning stage has been completed.

In summary, PP is a dynamic and integrative process essential for optimizing surgical outcomes. It requires a blend of intellectual rigor, technological support, and meticulous preparation to ensure that the surgical strategy is well-founded and that potential intraoperative challenges are anticipated and addressed.

### 2.2. Information Provided by 3D Data

(a) 3D CT scan

The acquisition of precise imaging data is a prerequisite for carrying out so-called “modern” PP. A 3D CT scan is the primary tool and the common base for the various 3D planning methods described later on. Specific information can be extracted from the DICOM images generated by a CT scan using specialized software. Different types of tissues (bone, soft tissues) can be identified through CT data segmentation, which relies on numerical algorithms and the radiological density of specific tissues. The transformation and classification of 3D images corresponds to the segmentation step [12]. For example, it can be used to view a single segment from a 3D-reconstructed limb. This 3D image, which is obtained through a fast automated process available in standard imaging software, is a PP method in itself. For example, this procedure is very useful for understanding and treating complex fractures in difficult trauma cases [13,14]. But, it remains a static plan, even though the 3D structure generated can be manipulated for better visualization. While CT data are used most often, 3D processing of imaging data generated by MRI and ultrasound may further enhance PP in the future [15,16,17].

(b) 3D modeling

Three-dimensional modeling provides considerable information that can be used for both dynamic and functional purposes. It is based on the processing of medical images by different algorithms with the aim of viewing, evaluating, and developing complex structures that reflect a patient’s anatomy. The software packages and techniques used for 3D modeling are grouped under the term “computer-aided design” (CAD).

The 1st application of CAD is the design of 3D objects. After the segmentation step, specific algorithms translate the spatial information associated with each voxel that makes up a 3D image into a specific geometry, made of points, lines, and surfaces—the result is represented in the shape of a triangle. The 3D model of the anatomical structure of interest is made from assembling multiple triangles. This model is now the base for the design of other 3D models, such as implants and guides specific to the patient’s anatomy, which can be used for the purpose of PP, but also for manufacturing (e.g., personalized implants) [18,19,20,21].

The 2nd application of CAD is 3D computer simulation. The behavior of systems can be simulated. Thus, based on the segmentation process, the various individualized tissues can be transferred to a CAD environment as 3D geometric entities and then used in another environment themselves. Finite element analysis software can be used to specifically study the physical behavior of each of the geometric entities after applying various mechanical constraints to them. Another type of software focused on detecting collisions can be used to study and simulate virtual movement between modelled objects. The latter application has recently been integrated into various PP software packages provided by implant manufacturers [22].

(c) 3D printing

Three-dimensional printing (or rapid prototyping or additive manufacturing) is a supplement to PP. Along with visual information, 3D printing allows a surgeon to manipulate an object reconstructed from the patient’s anatomy (complex fracture, bone deformity, tumor, etc.) or an instrument or implant designed specifically for a certain procedure (fracture fixation, osteotomy, etc.) [23,24]. These objects are fabricated from 3D digital files generated by CAD. Various additive manufacturing techniques can be used, depending on the material used and how it is transformed (stereolithography, fused deposition modeling, selective laser sintering, etc.). Various raw materials (textures) can be incorporated to reconstruct the material gradients unique to each structure [25]. One of the major advantages of this technology is that it is available to surgeons, who are no longer at the mercy of implant manufacturers, especially when the printed objects are solely for PP purposes, not tools that will be used during the surgery (guides, etc.…). But, the time needed to fabricate the parts, which is a function of their complexity, is a limitation of their routine use, especially in the context of planning for an urgent surgery in a trauma case.

(d) Mixed reality

**Figure 1:**
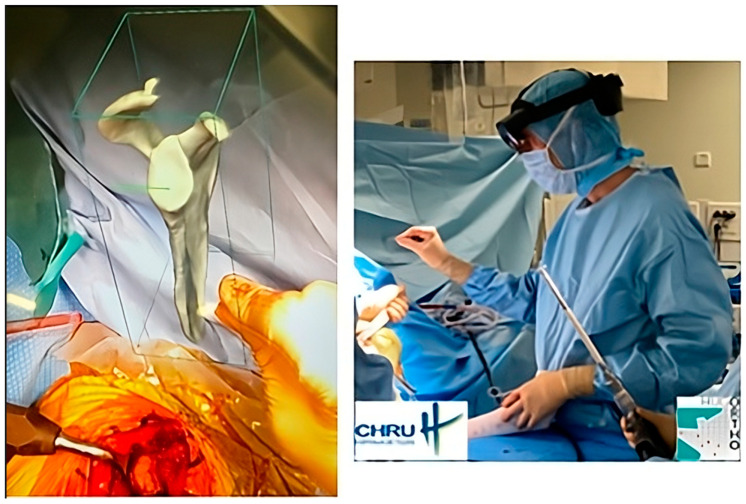
An example of intraoperative holographic visualization of a preoperative plan for a shoulder prosthesis, using the Blueprint™ 3D Planning software 4.0.2 (Tornier SAS, Montbonnot Saint Martin, France), viewed through an augmented reality headset. This visualization assists the surgeon in accurately placing the glenoid component during shoulder prosthetic replacement. The surgeon can refer to it at any point during the surgical procedure.

The most recent iteration of PP is virtual reality, which is a visualization method. Once completed, the plan is uploaded to optical see-through head-mounted displays or augmented reality (AR) glasses. Strictly speaking, this is not a planning method because the acquisition and 3D information creation steps are not performed with the data visualization support. Instead, AR provides a holographic plan that is visible and can be manipulated; the surgeon can refer to it at any point during the surgical procedure (Figure 1). But AR needs to become more robust (precise superimposition of virtual information and actual situation in real time) to improve PP data visualization, along with image-guidance methods [26,27,28].

### 2.3. Clinical Applications of 3D Planning

(a) Hip

Conventional 2D templating for hip replacement procedures, where implant models are superimposed over digital X-rays, is extensively used in clinical practice. It is widely accepted that the correspondence is accurate, and the reproducibility is good between the chosen implants and those planned on the 2D images. But, this method has several limitations: poor quality radiographs, magnification errors, inaccuracy when identifying the bony landmarks. Surgeon inexperience has also been identified as a limiting factor for the predictability of 2D templating [29].

The benefits of data visualization in the transverse plane provided by 3D PP have become obvious in the context of hip surgery. Other than the implant type and positioning being better suited to the patient’s anatomy, the joint biomechanics are better restored, with the surgeon’s experience having less of an impact. The measurement of leg length discrepancy is also more accurate and reproducible. A functional benefit was identified after observing hip replacement in difficult cases that incorporated 3D planning [30]. PP that resulted in a 3D-printed acetabular cup was useful for anticipating difficulties with implant positioning and for limiting the risk of dislocation during revision surgery [31,32,33].

(b) Knee

Three-dimensional PP has been shown to perfectly predict the size of personalized implants for knee replacement with up to 100% accuracy. This has the potential to improve intraoperative efficiency, minimize costs, and reduce surgical time. Additionally, 3D PP has been shown to be more accurate than 2D PP [34,35,36]. However, the accuracy of 2D appears to be sufficient to guide the choice of instrumentation needed to perform the knee replacement procedure. Ettinger et al. [34] compared 2D and 3D templating in 93 patients who underwent total knee arthroplasty. They found that 3D templating has very high accuracy for predicting the actual implant size, while 2D digital templating is accurate to within ±1 size for determining TKA components. They concluded that despite this slight discrepancy, 2D templating is still sufficiently accurate for launching template-directed instrumentation and does not require a high level of clinical experience from the examiner.

It is mainly in the context of osteotomy at the knee that 3D planning may be more relevant than conventional 2D methods [37,38,39,40], but with certain limitations: analyzing the deformity and corrections to perform in the various planes in space; choosing and positioning the fixation devices. Up to now, only one non-comparative study has been conducted on this topic. It reported accurate correction after standard tibial osteotomy planned in 3D using CT reconstruction software. However, the postoperative assessment in this study was performed using 2D radiographs [19]. There is currently no specific, validated protocol for 3D measurement of lower limb alignment [41].

(c) Shoulder

Three-dimensional PP can improve the analysis of glenoid deformities for shoulder replacement surgery, especially when extensive wear is present. It can also help with selecting implants and positioning them [42,43,44]. Conversely, no study to date has shown the benefits of using 3D planning instead of 2D in terms of the accuracy of the glenoid component implantation. The large variety of planning software currently available, which operate with different reference frames, brings into question whether the results can be compared and whether a gold standard should be defined [44,45,46]. Three-dimensional printing has also been used in the context of shoulder instability to recreate a glenoid defect, to analyze if humeral defect lesion comes into play, and how to best position the suture anchors [47,48].

(d) Spine

In a retrospective study, the functional outcomes of revision surgery for excision of a herniated disc were compared between two methods: one planned using 3D spinal models and the other conducted in the traditional manner, without planning. No differences in function were found between the two groups. However, surgery duration and blood loss were both lower in the planned surgery group [49]. Another recent study showed that three-dimensional printing improved surgical planning and the operational learning curve for endoscopic spinal surgery [50].

(e) Orthopedic oncology

**Figure 2:**
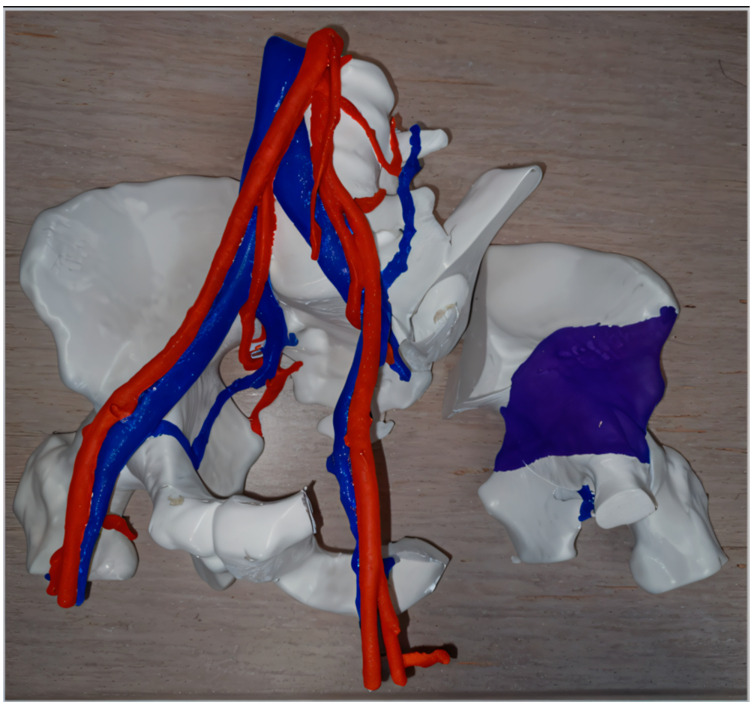
Example of preoperative planning by “independent” 3D printing of the resection of a chondrosarcoma in the left hemi-pelvis (planned resection margins in the right with purple color demonstrating the tumor) and its vascular relationships.

Three-dimensional printing has been used to simulate the resection of complex malignant tumors (Figure 2), allowing surgeons to better plan excision margins while protecting key vascular and nerve structures [26,51,52]. Intraoperative use of replicated tumor pieces that were sterilized—which allows surgeons to manipulate them—has been reported [24,53].

(f) Trauma

PP application through 3D printing in trauma cases ranges from reconstructing complex fractures for practice purposes, to determining which fixation devices to use and how to position them, with the devices themselves potentially being 3D-printed [23,54,55]. The 3D-printed parts can be used intraoperatively once they are sterilized. Printing of the healthy contralateral limb, to use as a “mirror image model”, can also supplement the planning of the desired fracture reduction [56].

## 3. What Does Modern Motion Analysis Consist of?

### 3.1. Principles and Objectives of Motion Analysis

Movement is the result of an interaction between multiple anatomical systems (neurological, muscular, skeletal). These systems must be intact for movement to achieve a defined objective in a given environment. If one of these systems is altered, movement becomes difficult, painful, or even impossible. Gaining a full understanding of the abnormal movement requires a precise analysis of the dysfunction itself and the various structures (particularly anatomical ones) that may be contributing to this dysfunction.

Motion analysis starts during the in-office consultation by determining the patient’s symptom history and carrying out a comprehensive physical exam, which may include photographs or video. Questionnaires about function and simple measurement tools (goniometer, dynamometer, either manual or smartphone applications) are used. This standard approach to motion analysis is mainly a “passive”, static, and 2D one (evaluation and grading of a joint’s single degree of freedom in a given plane). In the end, it is not very accurate and is not very reproducible [10,57]. More accurate and complex motion analysis methods exist, but they are mainly used in research laboratories. These approaches are more “active”, dynamic, and are in 3D.

Depending on the measurement objectives, motion analysis can be grouped into three domains:

− The first is kinematics. This integrates methods of joint or segmental motion analysis, i.e., analysis of the movement amplitude of two articulated bone segments caused by muscles, typically while using surface markers. It also consists of measuring spatiotemporal aspects, for example, while a patient walks on a treadmill.
− The second is kinetics, which encompasses methods that evaluate the forces occurring during movement, joint moments, and applied loads. Force platforms and dynamometers are used.
− The final is electromyography, which consists of measuring deep or superficial muscle activity, at rest and during movement. Electromyography can be used to evaluate the functional link that influences the other movement parameters measured by kinematics and kinetics methods.

### 3.2. Modern Analysis Methods for Joint Movements

(a) Video analysis

Video analysis is the simplest and least costly. It can identify obvious abnormalities during movement (e.g., walking). A more detailed analysis can be performed by slowing the video or stopping on certain images. But, this returns it to being a static analysis and is limited to a 2D reference frame. The accuracy and reliability of angle measurements are questionable, given the distortion of images captured. While acquiring data with a standard video camera is simple, a detailed experimental protocol is needed (movement execution, filming points, etc.). The use of goniometers integrated into a smartphone has started to replace this type of visual movement analysis, as it provides a simple joint range of motion (ROM) measurements [58,59].

(b) Optoelectronic motion analysis

**Figure 3:**
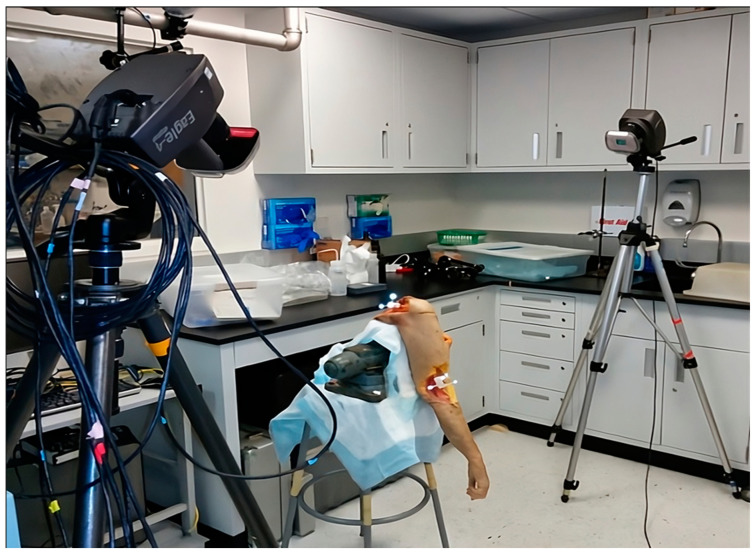
Setup for an optical motion analysis system using cameras that emit and detect infrared signals along with optoelectronic markers secured to an upper limb cadaver specimen (Motion Analysis Laboratory, HSS, New York, NY, USA).

This method consists of tracking the position of passive reflective markers (so-called optoelectronics) on the skin’s surface, typically over prominent anatomical landmarks, on either side of the joint of interest, with known degrees of freedom. The limb segments are also defined by markers around the articulation that joins them. Several cameras emit infrared rays, which are reflected by the various markers and sent back to these cameras. After the processing of images from at least two cameras, the positioning of limb segments can be deduced and reconstructed by triangulation in the 3D recording space. Thus, by using these infrared data and pairing them with specific software, it is possible to analyze movements in 3D for the (recreated) joint of interest. An example of optoelectronic motion analysis is demonstrated in Figure 3.

The main limitation of this method is micromovement of the markers due to soft tissue gliding, which varies depending on the subjects and the types of movements being studied. Deformation of the reflected signal, potential marker occlusion, and marker identification are other technical limitations. Thus, the reliability and accuracy of these measurements are also questionable, especially when measuring joints with small ROM, such as the knee, in the “Axial” plane [60].

(c) Markerless motion capture

**Figure 4:**
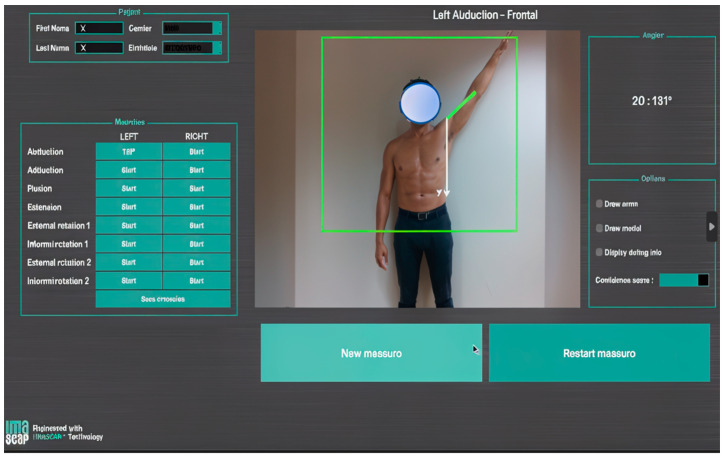
Screenshot from a motion analysis software that uses markerless capture with an RGB-D camera augmented by an AI algorithm, which was used during an in-office consultation (provided with permission from Gauci, IULS, CHU Nice; Imascap).

This initially consisted of capturing an individual’s silhouette (silhouette tracking) with several conventional 2D cameras and then using this information to construct a 3D model [61]. This model is then decomposed into several limb segments that are articulated with each other. The movements of the selected segments can then be recorded and analyzed in every plane. These various steps may require several software interfaces. The devices being used have now become lighter and simpler. They are based on the emission of an infrared light towards the patient, whose reflected image is captured and then processed to reconstruct information about depth and, consequently, the third dimension. This is made possible by a unique RGB-D camera that couples color and depth information (RGB: color, D: depth) and the development of algorithms that can optimize its functioning [62]. This is a major technical advance because surface markers are no longer needed, which makes this technology easier to use in a clinic or office (Figure 4). This technology provides fast measurements of joint ROM, with a similar accuracy to photographic captures [63,64].

(d) Connected devices

The emergence of connected devices and, more specifically, connected implants provides a new avenue for motion analysis. Up to now, this type of functional analysis has mostly focused on total knee arthroplasty. Nevertheless, the technical challenges are daunting because the system must acquire data that are “active”, continuously or transiently, and their reliability depends on the sensors used. Along with being accurate, the sensors must be miniaturized, self-powered, sturdy, water-tight, corrosion-resistant, and able to communicate with the exterior for the recording and processing of the data captured. The new format of these data makes for complex secondary analysis [65].

(e) Augmented reality headset

There have been recent reports of an AR headset being used for motion analysis during walking. The principle is to combine motion capture by using a camera integrated into the headset to a holographic display of footprints that serve as a guide on the ground of the target gait pattern, which is then recorded. The analysis focused on the joint ROM and the difference between the true footsteps and the holograms. Nevertheless, optoelectronic markers are still needed for this application [66]. Additional information by haptic feedback can also be recorded to evaluate the differences in the stress distribution due to an alignment defect in the lower limbs [67]. An AR headset might be used in combination with connected devices in the not-too-distant future.

### 3.3. Limitations of Current Motion Analysis Methods

The main limitation of these motion analysis methods is data interpretation. There are considerable inter- and intra-individual variations when performing a movement. The reference kinematic values (mean, SD) for a given movement are generally established from values measured in a healthy adult population, selected based on specific criteria. However, this does not mean that individuals whose results fall outside of the reference range have an abnormal or pathological movement. Any difference relative to reference values must be interpreted carefully, in the proper context, and in concertation with the specialist performing the motion analysis. This limitation is already daunting when performing a functional evaluation in a healthy population—it will be even greater when analyzing movement in a pathological joint [68].

The large range of methods currently being used for motion analysis and the lack of an accepted gold standard are additional limitations for the interpretation and comparison of results. Thus, it is difficult to conclude that one motion analysis method is better than the others. We should focus on measurement reproducibility to determine whether an analysis method is relevant. Within a single method, reproducibility is highly sensitive to protocol variations (e.g., patient’s position), the analysis plane used for a given movement, and the type of joint being studied [69].

## 4. How Can Preoperative Planning and Functional Movement Analysis Be Connected?

### 4.1. From Morphological to Functional

The challenge lies in matching PP based on an implant’s position, with the surgical goal of functional recovery. The criteria for good-quality statically planned implant positioning do not always correspond to the conditions for optimal implant functioning [70] nor, consequently, the appropriate functional outcomes for a given patient. The collection, use, and analysis of perioperative data with the integration of AI is likely one of the main research avenues for establishing a more successful model for predictive functional planning.

### 4.2. Definition of Artificial Intelligence

According to the Cambridge Dictionary, artificial intelligence corresponds to “computer technology that allows something to be done in a way that is similar to the way a human would do it”. By extension, it designates, in current language, the devices that imitate or replace humans in certain implementations of their cognitive functions. Computer programs are now used to carry out tasks typically performed by humans but that require high-level mental processing (perceptual learning, critical reasoning, memory organization) and could be fallible if one is not a subject matter expert [71,72].

### 4.3. Role of Artificial Intelligence

(a) Clinical functional analysis

AI has been used to identify prognostic factors of functional outcome after various types of arthroplasty [73,74,75,76]. The potential application of AI in large databases is certainly exciting, as it might allow us to identify predictive factors that have not yet been identified by traditional statistical methods in smaller populations [77]. Functional outcomes (scores, joint ROM) have been successfully predicted after the application of various learning algorithms that were first trained with preoperative data [78,79]. New functional scores, constructed from the predictive factors identified by AI, have been proposed for the postoperative clinical evaluation of shoulder replacements. One of the advantages of these scores is that they better-capture patient expectations [80]. The tendency is to be increasingly selective, personalized, and, therefore, accurate in choosing which postoperative functional evaluation parameters will be used for a given surgical procedure [81].

(b) Motion analysis

AI algorithms can contribute to motion analysis on two levels. The first is interpreting the captured data. For example, with machine learning models, the contribution of various joints involved in a movement during its successive phases can be decomposed [82,83,84]. The second is the improved precision that can be achieved by coupling an AI algorithm with a markerless motion capture system [85,86]. This type of software interface, based on a deep learning algorithm, helps to get around imperfections when recording a movement with an RGB-D camera, and thereby to improve the measurement accuracy. This bodes well for the analysis of complex movements, combined in several planes, and in an outpatient setting. The development of these new AI algorithms will also contribute to the use of new functional capture methods, such as AR headsets.

(c) Preoperative planning

AI may contribute to improving the accuracy and, especially, the predictive ability of PP methods [87,88]. Machine learning algorithms can be used with CAD models of the different elements and steps needed to construct PP software (bone geometry, soft tissues, classification, material behaviors, collision detection, etc.). Other training algorithms can be applied to the planning software itself to increase its robustness and predictive power, thereby mimicking the real conditions of the operation, and also its potential outcome [89,90,91]. There may come a time where a preoperative plan is proposed to a surgeon thanks to an AI algorithm constructed based on feedback from a panel of expert surgeons.

### 4.4. Limitations of Artificial Intelligence

The primary limitation of AI is the quality of the input data needed for machine learning. While it is possible to extract various data retrospectively from registries [92], their quality is typically not sufficient if they were not put into place when the patient’s care started. The functional data must be reproducible, defined precisely, and collected prospectively. This affects their comparability before and after surgery, and their reliability when included in a predictive model. The other limitation of AI is the lack of external validation of the machine learning algorithms currently used in orthopedics [93,94].

## 5. What Is Still Missing to Achieve Predictive Functional 4D Planning?

### 5.1. Soft Tissues

**Figure 5:**
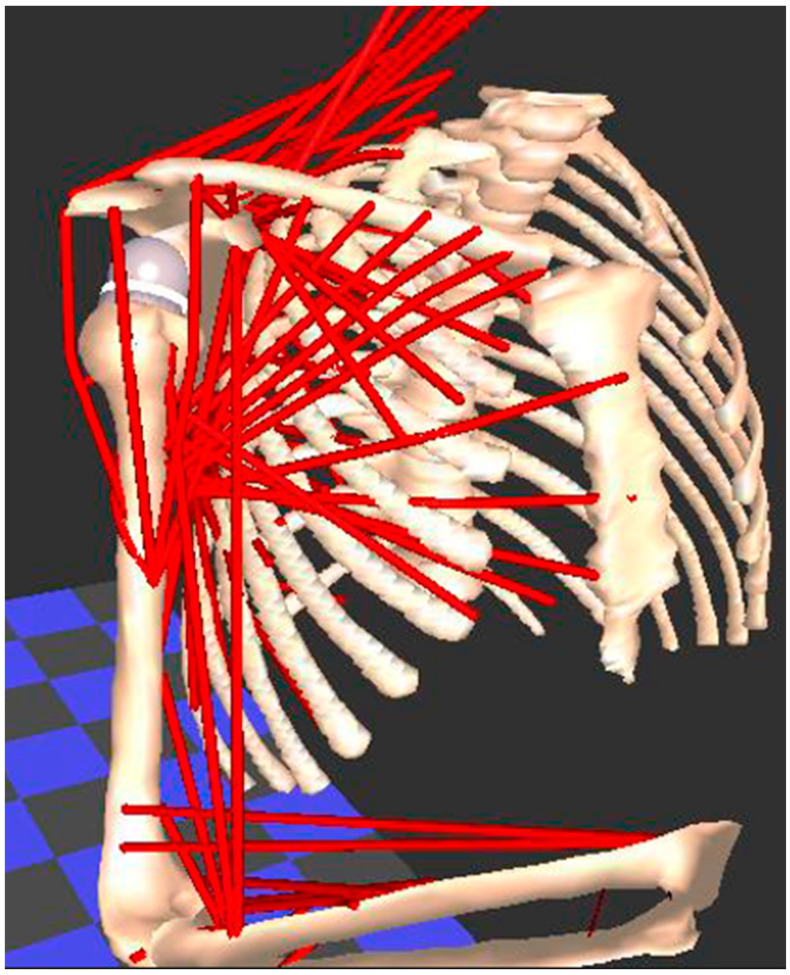
The Newcastle shoulder model used for biomechanical analysis of the muscles in the upper limb. Each muscle is modelled as one or more red “lines”, whose change in length and moment are calculated when a movement is applied to the model.

Evaluating soft tissues (muscle, tendon, ligament, cartilage) is the weak link in every motion analysis method that has been previously described. However, this is a basic tenet for understanding complex freely moveable joints, such as the shoulder and hip, which function in a similar way. Both are ball-and-socket joints with multiple degrees of freedom, which increases the risk of instability due to bone impingement or muscle imbalances. This muscle behavior, in passive and active scenarios, marked by a certain deformability between elongation and shortening, corresponds to information about relaxation and contraction, which is difficult to measure in practice, especially during complex movements. Physical and computerized muscle models have been developed for measuring muscle length and moment arms in healthy individuals [95,96,97,98]. Figure 5 demonstrate an example of a biomechanical analysis of the muscles in the upper limb using the Newcastle shoulder model [99].

### 5.2. Defining the 4D Concept

In relativity, the fourth dimension represents the time portion of the space–time continuum. Starting with the coordinates of a 3D object (x, y, z), another coordinate is added—called t or w—which is a new direction, perpendicular to all the directions in space. With this coordinate, 4D introduces the notion of movement or transformation applied to a 3D object, which can “move” relative to its initial state.

### 5.3. Towards Functional 4D Planning

An application of the 4D concept has already been reported in the context of predictive PP, where the deformation of “rigid” 3D structures (bones and implants) was analyzed [100]. Similar applications could be developed based on volumetric 3D muscle models. The muscles’ capacity for excursion and lengthening around bony segments during a movement could be measured, thereby evaluating their passive participation. Its integration into planning software would be a crucial step toward making the functional prediction more accurate.

### 5.4. Using a Functional 4D Plan

(a) Hip

Several parameters related to functional outcomes after total hip replacement have now been integrated into PP software (ROM, mechanical impingement, overhang of acetabular cup, leg length, etc.). However, these are typically analyzed individually, not in combination. It only indirectly translates the effect of joint replacement on muscle balance in the hip, and its good theoretical function. But, the analysis of these parameters depends on the choice and positioning of the implants, which are specific and optimized for a given patient and his/her anatomy. Recently, the concept of a “patient-specific combined target zone” (PSCTZ) has been described [101,102]. It consists of identifying (for a given patient) the implant position that will produce the best result possible in all the functional PP parameters described previously. Clinically, the patients whose implants were placed in the PSCTZ had a higher Harris Hip Score than the those patients for whom the implants were in the conventional “safe zones” (Dorr or Lewinek) [103]. Through this digital–clinical correlation, a new gold standard for implant positioning was defined. The next step could be to analyze the choice and positioning of implants corresponding to the PSCTZ in patients who had the best functional outcome. A further step could be to investigate how the periarticular muscles (length, moment arm, etc.) are affected by the various implant configurations available (offset, length, etc.). The muscle data on the operated side could be compared with the healthy contralateral side, which is used as a reference and as the functional biomechanical goal [104]. In the end, all these data could be integrated into an advanced planning software package and could form the basis for functional 4D simulation.

(b) Shoulder

Functional 4D planning models for the shoulder already exist [105,106]. They are similar to the creation of a digital twin, which combines various CAD methods as a function of the information sought during PP. Starting with a cadaver specimen and optoelectronic markers, CT data are acquired, and motion is captured before and after shoulder replacement implants are added. This entire dataset is then integrated into CAD software (motion analysis, finite elements, etc.). Various implant configurations can be tested relative to the functional objectives and the available muscle data.

More recently, muscle segmentation using CT scan data from patients has been described [107,108]. Volumetric information, differentiating “efficient” muscles from ones infiltrated with fatty tissue, were analyzed based on primary or secondary shoulder degenerative conditions. This type of information, when paired with clinical patient data or incorporated into other CAD models, paves the way for personalized functional planning.

## 6. Conclusions

Modern PP corresponds to accurate planning based on the segmentation of 3D images, which are used for visualization and modeling. Despite proven accuracy and current advantages, the clinical benefit of 3D planning has not yet been demonstrated clearly, as it is still in its early stages. The lack of reference values makes it difficult to carry out studies comparing the various planning methods.

Modern motion analysis must combine the functional data from clinical observation with more accurate data from dynamic and 3D motion capture. We can now perform motion analysis in our offices, with thanks to the advent of markerless capture systems and appropriate software packages.

The goal of combining PP with motion analysis would be to develop a model that successfully predicts functional outcomes after surgery. This application of AI has the potential to generate valuable predictive models, as long as the input data are accurate and the machine learning algorithms are validated.

Improvement in muscle modeling, combined with AI, opens the door for functional, so-called, 4D planning, which should be even better at predicting the outcomes of surgery. Together, these technological advances may eventually result in dynamic, functional, and personalized PP.

## Data Availability

The data presented in this study are available upon request from the corresponding author.
